# Complete Genome Sequence of Pseudomonas aeruginosa Bacteriophage vB_PaeP_PaCe

**DOI:** 10.1128/mra.00463-22

**Published:** 2022-07-18

**Authors:** Ryan Cook, Taylor Darby, Dwayne R. Roach

**Affiliations:** a School of Veterinary Medicine and Science, University of Nottingham, Loughborough, Leicestershire, United Kingdom; b Department of Biology, San Diego State University, San Diego, California, USA; c Viral Information Institute, San Diego State University, San Diego, California, USA; Queens College CUNY

## Abstract

Here, we report the complete genome sequence of the virulent podovirus PaCe, which was isolated from wastewater in San Diego, California, using the host Pseudomonas aeruginosa. Its complete genome is 45,365 bp in length, with a GC content of 52.5%. PaCe belongs to the genus *Bruynoghevirus* in the class *Caudoviricetes*.

## ANNOUNCEMENT

Bacteriophages are the predominant biological entities on the planet. The recent increase in sequence information has shed some light on their diversity, although much diversity remains unseen (1). Uncovering new phages with human and animal therapeutic potential also represents important discovery (2–4). Here, we describe the genome of a new Pseudomonas phage, PaCe (vB_PaeP_PaCe), which was isolated from a single plaque after enrichment in Luria broth using a defined O-antigen polysaccharide mutant of Pseudomonas aeruginosa strain PAO1 and was purified using five consecutive serial dilutions.

PaCe DNA was isolated using phenol-chloroform extraction, and sequencing libraries were prepared using the Nextera XT kit. The libraries were sequenced on an Illumina NextSeq 550 instrument (2 × 150-bp paired-end reads) at the Microbial Genome Sequencing Center (Pittsburgh, PA), and the resulting reads were processed as described previously (5). In brief, adapter sequences and reads mapping to ΦX174 were removed from the 11,758,880 raw reads using BBDuk.sh v38.69, followed by trimming with the following parameters: ktrim=r hdist=1 tpe tbo minlen=100 qtrim=rl trimq=28 (6). The remaining reads were quality controlled using FastQC v0.11.5 (https://www.bioinformatics.babraham.ac.uk/projects/fastqc) and assembled into a single contig using SPAdes v3.12.0 with default parameters (7). Termini could not be predicted using PhageTerm (8); however, the genome was found to be circularly permuted using apc.pl (https://github.com/jfass/apc), and the repeated sequence artifacts were removed. The PaCe genome was manually reordered to match the most closely related phage based on average nucleotide identity, Pseudomonas phage Epa4 (GenBank accession number MT118288), as determined using the get_closest_relatives.pl script as part of the INPHARED suite (1). The final assembly had a median coverage of 15,199-fold, as determined by BBMap.sh v38.69 with default parameters (6). Annotation was performed using Prokka v1.14.6 with hidden Markov models (HMMs) produced from Prokaryotic Virus Remote Homologous Groups (PHROGs) (http://s3.climb.ac.uk/ADM_share/all_phrogs.hmm.gz), a publicly available Prokka database of bacteriophage annotations curated by the Millard laboratory (http://s3.climb.ac.uk/ADM_share/crap/Caudovirales.tar.gz), the phage Epa4 GenBank entry (GenBank accession number MT118288), and the well-characterized *Bruynoghevirus* phage LUZ24 GenBank entry (GenBank accession number NC_010325) (9, 10). The complete circularly permuted genome of PaCe is 45,365 kb in length, with a GC content of 52.5% and a coding density of 89.6%. Annotations predict 73 coding features, including four tRNAs (Pro, Tyr, Asp, and Asn). The genome lacks known lysogeny genes using a set of HMMs, suggesting that PaCe has a strictly lytic lifestyle (11). Furthermore, no virulence factor or antibiotic resistance genes were identified using Abricate with ResFinder and the Virulence Factor Database (VFDB) (12–14).

PaCe displays genome-wide similarity to 40 other phages in the genus *Bruynoghevirus* in the class *Caudoviricetes*, sharing 97.1% nucleotide identity with Pseudomonas phage Epa4 (GenBank accession number MT118288) and Pseudomonas phage PaP_Se (GenBank accession number OL441337), 96.4% nucleotide identity with Pseudomonas virus LUZ24 (GenBank accession number NC_010325), and 89% nucleotide identity with Pseudomonas phage Epa1 (GenBank accession number MT108723). Furthermore, PaCe was found to encode the novel DNA gyrase inhibitor peptide Igy, which was recently described for LUZ24 (15).

### Data availability.

The PaCe genome is available in GenBank with accession number ON376263. Sequencing reads are part of the Sequence Read Archive (SRA) under BioProject accession number PRJNA833929 and BioSample accession number SAMN28024470.
